# Microvascular and oxidative stress responses to acute high-altitude exposure in prematurely born adults

**DOI:** 10.1038/s41598-023-34038-6

**Published:** 2023-04-26

**Authors:** Giorgio Manferdelli, Benjamin J. Narang, Vincent Pialoux, Guido Giardini, Tadej Debevec, Grégoire P. Millet

**Affiliations:** 1 Institute of Sport Sciences (ISSUL), University of Lausanne, Synathlon, 1015 Lausanne, Switzerland; 2 Department of Automatics, Biocybernetics and Robotics, Jožef Stefan Institute, Ljubljana, Slovenia; 3 Faculty of Sport, University of Ljubljana, Ljubljana, Slovenia; 4 Institute of Biostatistics and Medical Informatics, Faculty of Medicine, University of Ljubljana, Ljubljana, Slovenia; 5 Laboratoire Interuniversitaire de Biologie de La Motricité UR 7424, Faculté de Médecine Rockefeller, Univ Lyon, Université Claude Bernard Lyon 1, 69008 Lyon, France; 6 Institut Universitaire de France, Paris, France; 7 Mountain Medicine and Neurology Centre, Valle D’Aosta Regional Hospital, Aosta, Italy

**Keywords:** Biochemistry, Physiology

## Abstract

Premature birth is associated with endothelial and mitochondrial dysfunction, and chronic oxidative stress, which might impair the physiological responses to acute altitude exposure. We assessed peripheral and oxidative stress responses to acute high-altitude exposure in preterm adults compared to term born controls. Post-occlusive skeletal muscle microvascular reactivity and oxidative capacity from the muscle oxygen consumption recovery rate constant (*k*) were determined by Near-Infrared Spectroscopy in the *vastus lateralis* of seventeen preterm and seventeen term born adults. Measurements were performed at sea-level and within 1 h of arrival at high-altitude (3375 m). Plasma markers of pro/antioxidant balance were assessed in both conditions. Upon acute altitude exposure, compared to sea-level, preterm participants exhibited a lower reperfusion rate (7 ± 31% vs. 30 ± 30%, *p* = 0.046) at microvascular level, but higher *k* (6 ± 32% vs. −15 ± 21%, *p* = 0.039), than their term born peers. The altitude-induced increases in plasma advanced oxidation protein products and catalase were higher (35 ± 61% vs. −13 ± 48% and 67 ± 64% vs. 15 ± 61%, *p* = 0.034 and *p* = 0.010, respectively) and in xanthine oxidase were lower (29 ± 82% vs. 159 ± 162%, *p* = 0.030) in preterm compared to term born adults. In conclusion, the blunted microvascular responsiveness, larger increases in oxidative stress and skeletal muscle oxidative capacity may compromise altitude acclimatization in healthy adults born preterm.

## Introduction

Preterm birth affects over 10% of live births worldwide, and those born moderately to extremely preterm (≤ 32 weeks gestation) are considered at highest risk for short- and long-term respiratory and cardiovascular morbidity^1^. Though a growing number of preterm birth survivors reach adulthood, increasing evidence supports several long-term sequelae of early delivery on the cardiovascular system^2,3^, reducing exercise capacity^4^ and ultimately leading to increased cardiovascular and metabolic disease risk^5^. Given that several of these comorbidities have been associated with mitochondrial dysfunction and chronic oxidative stress^6^, it is reasonable to hypothesize that premature birth evokes specific peripheral responses to certain stimuli^7^.

Previous attempts to investigate vascular health in young adults born preterm have provided equivocal results. For example, endothelial function assessed by flow-mediated dilation or finger plethysmography was reported as normal^5,8^ or reduced^2,9^. In contrast, both microvascular function and density were found to be reduced in adults born prematurely^3^.

Premature birth also seems to impact redox balance, likely as a consequence of perinatal supplemental oxygen therapy^10^. However, the results of these investigations are also not consistent. Systemic oxidative stress markers have been reported to be higher (e.g., 8-isoprostane in exhaled breath condensate, and total superoxide dismutase (SOD) and glutathione peroxidase (GPX) in blood)^10^ or similar (e.g., advanced oxidation protein products (AOPP), Catalase and SOD in plasma)^11^ in preterm adults compared to term born peers. Given that oxidative stress is known to be modulated by environmental hypoxia^11^ and, simultaneously, plays a central role in the individual response to hypoxia^10^, a specific phenotypical response to hypoxia may be present in prematurely born adults. However, to date, findings regarding the systemic and localized effects of acute hypobaric hypoxia on redox balance modulation and microvascular reactivity in this population remain scarce.

Oxidative stress is also known to be a potent modulator of oxidative capacity and metabolic responses at the skeletal muscle level in adults born preterm^10^. Animal models used to simulate premature birth by postnatal hyperoxic exposure have demonstrated alterations in muscle and mitochondrial functions^12^. These finding where recently extended to adult humans born prematurely, where mitochondrial function in peripheral blood mononuclear cell was increased compared to control peers born at term^13^. However, despite these preliminary findings, no in vivo evidence on skeletal muscle oxidative function is currently available in preterm individuals.

Near-infrared spectroscopy (NIRS) in association with vascular occlusion testing (VOT) has recently emerged as a non-invasive method of evaluating resting muscle oxygen consumption and microvascular reactivity^14,15^, as well as muscle oxidative capacity^16–18^. A blunted microvascular hyperemic response following an ischemic stimulus has been previously reported in diseased populations with cardiovascular impairments^19^ and in sedentary (relative to endurance-trained) individuals^20,21^. Similarly, skeletal muscle oxidative capacity was found to be significantly lower in patients with pulmonary^22^ and cardiovascular diseases^23^; conditions associated with reduced exercise capacity. A recent mechanistic study assessed skeletal muscle O_2_ diffusion capacity (DmO_2_) by evaluating skeletal muscle oxidative capacity under high and low levels of tissue O_2_ availability in the *vastus lateralis* muscle^18^. This technique was subsequently proposed to also be applicable with the use of systemic hypoxia; a decreased whole-body O_2_ supply was proposed as a surrogate for the low oxygen availability conditions that allow DmO_2_ computation^24^.

Accordingly, the aim of this study was to investigate the acute peripheral (microvascular reactivity and skeletal muscle oxidative capacity) and systemic oxidative stress responses to high-altitude exposure in healthy prematurely born adults and their age-matched term born peers. We tested the hypotheses that (i) preterm adults would demonstrate impaired microvascular reactivity and skeletal muscle oxidative capacity, and that (ii) acute high-altitude exposure would reduce these impairments relative to their term born peers.

## Methods

### Participants

Thirty-four young healthy men volunteered and gave written informed consent to participate in this study. Seventeen participants were born preterm. Participants were matched for age and fitness status. All participants were not taking any medication and were free from any cardiorespiratory, neurological and hematological diseases. Moreover, none of the participants was a habitual smoker. The preterm born participants were recruited via the National preterm birth register managed by the University Clinical Centre in Ljubljana, Slovenia using medical record screening and telephone/email-based individual interviews. Importantly, the included preterm participants had a gestational age ≤ 32 weeks, a birth weight ≤ 1500 g, had received postnatal supplemental oxygen therapy, and none of the participants had a history of bronchopulmonary dysplasia. The inclusion criteria for the term born participants were gestational age ≥ 38 weeks and birth weight ≥ 2500 g. Birth-related inclusion criteria for all participants were checked and confirmed during the initial birth/medical record screening procedure conducted prior to inclusion in the study. The experimental protocol was pre-registered at ClinicalTrials.gov (NCT04739904), approved by both the University of Ljubljana, Faculty of Sport ethics committee (8/2020-316) and the Aosta Hospital Ethical Committee (06/05/2021.0038781.I), and performed in line with the Declaration of Helsinki guidelines.

### Experimental design and ascent protocol

Each participant underwent two experimental trials, the first one near sea-level (Ljubljana, Slovenia; barometric pressure ~ 737 ± 0.5 mmHg) and the other one within 1 h of arrival at high-altitude (3375 m; Torino hut, Aosta Valley, Italy, on the Mont Blanc massif; barometric pressure ~ 503 ± 0.7 mmHg). Subjects traveled to Courmayeur (1300 m) by car, then traveled by cable car to Torino hut in 15–20 min. On average, the duration between the two phases was 97 ± 4 days. By performing the sea-level measurements prior to the high-altitude exposure, we ensured that there were no potential carryover effects of altitude testing on the subsequent control tests, as would have been the case for some participants in a randomized crossover design. During the informed consent visit, participant performed a cycling exercise test and a 5-min leg occlusion to familiarize with the testing procedures. Blood pressure was measured on the left arm at rest both at sea-level and upon arrival to altitude using a digital sphygmomanometer (M2, OMRON Healthcare, Hoofddorp, The Netherlands).

#### NIRS-VOT-derived muscle oxygenation and reactivity

At sea-level and upon arrival at altitude, participants rested in seated position on a cycle ergometer before performing a VOT consisting of 5 min of tissue ischemia via femoral artery occlusion, followed by 5 min of vascular reperfusion^15^. To ensure signal stability, tissue ischemia was only initiated after tissue saturation index (TSI) had been stable for 30 s (< 2% variation). Occlusion was accomplished using a pneumatic cuff placed on the proximal part of the thigh and connected to an automatic rapid inflation system (HokansonE20 AG101, Bellevue, WA, USA). Occlusion pressure was set between 290 and 300 mmHg and maintained for the full 5-min period of ischemia. Cuff pressure was identical at sea-level and at altitude. Oxygenation changes in the *vastus lateralis* muscle were evaluated by a continuous wave NIRS device (Portamon, Artinis Medical Systems, Elst, The Netherlands), which consisted of three dual-wavelength (760 and 850 nm) light transmitters-channels and one receiving optode. Three distances (30, 35 and 40 mm) were adopted between the receiver and transmitters. The NIRS probe was placed on the lower third of the *vastus lateralis* muscle (~ 10 cm above the knee joint) of the right thigh. The skin overlying the investigated muscle region was carefully shaved before experimentation, and the same experimenter placed the probe to minimize the variability in positioning between tests. The probe was secured to the skin using double-sided tape, and elastic bandages were wrapped around the probe and the thigh to avoid ambient light contamination. Adipose tissue thickness (ATT) at the site of the NIRS probe was measured using a skinfold caliper. TSI changes were monitored during the test. NIRS data were recorded at 10 Hz and exported at 5 Hz. Baseline TSI (%) was calculated as the 30 s average before cuff occlusion. As previously proposed^15,21^, the linear regression of TSI signal during the first minute of occlusion (desaturation rate, % s^−1^) was taken as an index of resting skeletal muscle oxidative metabolism. The rate of reperfusion (reperfusion rate, % s^−1^) was quantified as the upslope of the TSI signal during the first 10 s following cuff release. Minimum and peak TSI (TSI_min_ and TSI_peak_, respectively, %) were calculated as the lowest and highest recorded TSI value during ischemia and reperfusion, respectively, and their difference was used to compute the amplitude of the change (A_TSI_, %). The time required for the TSI signal to return to baseline values after cuff release (*t*_*baseline*_) was also evaluated. The area under the curve for the TSI signal (AUC_2MIN_) was calculated from the reperfusion overshoot (area under the reperfusion curve above baseline until 2-min post cuff release).

#### Skeletal muscle oxidative capacity

After having checked that the TSI signal following VOT returned to baseline values (~ 5 min), skeletal muscle oxidative capacity was evaluated in vivo by measuring post-exercise muscle oxygen consumption (*m*V̇O_2_) recovery kinetics by NIRS and the repeated occlusions method^16,17^. The protocol consisted of a 3-min rest followed by a 10-min moderate intensity (~ 50% V̇O_2peak_) constant work rate exercise bout aiming to increase *m*V̇O_2_ and desaturate the muscle to ~ 50% of A_TSI_ (i.e. the physiological range)^18,22^. The exercise bout was immediately followed by a series of 5-s intermittent arterial occlusions (5 separated by a 5-s cuff release, 5 separated by a 10-s release, and 5 separated by a 20-s release). As per VOT, occlusion pressure was set between 290 and 300 mmHg and it was kept the same between the two conditions. For each intermittent arterial occlusion, the rate of decline in TSI (expressed in % s^−1^) was fitted by a linear function to estimate relative *m*V̇O_2_. Importantly, as previously reported^22^, during arterial occlusion the deoxygenation rate is inversely proportional to *m*V̇O_2_, and it is therefore reported below as a positive value (%s^−1^). *m*V̇O_2_ values were then fitted by a monoexponential function of the type:

$$y\left( t \right) = y_{END} - Delta \times e^{ - 1/\tau }$$

where, y(t) represents the *m*V̇O_2_ value at a given time (t), y_END_ the *m*V̇O_2_ value immediately after exercise cessation, Delta is the change in *m*V̇O_2_ from rest to the end of exercise, and τ is the rate constant (*k* = [1/τ], expressed in min^−1^) of the function. The exponential *m*V̇O_2_ recovery rate constant (*k*, min^−1^) was estimated using nonlinear least-squares regression and was taken as an estimate of skeletal muscle oxidative capacity. As previously proposed^18,24^, DmO_2_ was estimated as the absolute difference between *k* obtained under normoxic and hypoxic conditions.

### Plasma pro-oxidant/antioxidant balance

#### Blood sampling

At sea-level and upon arrival at altitude, 6 mL of venous blood were obtained from the antecubital vein with the participants in a seated position. Blood samples were drawn into ethylenediaminetetraacetic acid blood collection tubes and centrifuged (10 min at 3500 rpm, 4 °C). Subsequently, the obtained plasma was aliquoted into three 1.5 mL cryotubes, which were immediately frozen to −80 °C until analysis^25^.

#### Biochemical analyses

All spectrophotometry and fluorometry measurements were performed with the TECAN Infinite 2000 plate reader (Männedorf, Switzerland).

#### Oxidative stress markers

AOPP levels were measured via spectrophotometry by reading at 340 nm 40 μL of plasma diluted in 200 μL of PBS 1X with 20 μL of acetic acid (99–100%) in 96-well microtest plates. AOPP level was computed using chloramine-T standard solution, which absorbs at 340 nm in the presence of potassium iodide.

Xanthine oxidase (XO) activity was determined by measuring the appearance kinetics of the complex superoxide anion and nitrotetrazolium blue (NTB) by spectrophotometer at 560 nm for 10 min.

Similarly, myeloperoxidase (MPO) activity was measured by a semi-quantitative immunoassay using stabilized human anti-MPO antibodies (MPO, Human, clone 266-6K1, HM2164, Hycult Biotech). The MPO/anti-MPO complex was detected by spectrophotometry after addition of a 3,3’,5,5’-tetramethylbenzidine solution (TMB, Sigma) with H_2_O_2_ as a chromogenic substrate.

#### Antioxidant enzymes

Catalase activity was determined by measuring the kinetics of formaldehyde apparition formed by the reaction between methanol and hydrogen peroxide (H_2_O_2_), which is catalyzed by catalase. 30 μL of methanol (100%) and 20 μL of H_2_O_2_ solution (0.14%) were added to 20 μL of plasma diluted in 100 μL of PBS 1X in 96-well microtest plates. After 20 min the reaction was inhibited by adding 30 μL of a potassium hydroxide solution (10.69 mol·L^−1^). Formaldehyde was revealed by adding 30 μL of purpald solution (0.20 mol·L^−1^), and its concentration was measured 5 min later by spectrophotometry at 540 nm and computed using formaldehyde standards.

GPx activity was assessed by measuring nicotinamide adenine dinucleotide phosphate hydrogen (NADPH) consumption, which is proportional to GPx activity to reduce H_2_O_2_ in the presence of glutathione reductase and reduced glutathione. Glutathione reductase, NADPH (10 mmol·L^−1^) and reduced glutathione solutions were added to 20 μL of plasma diluted in 200 μL of PBS 1X in 96-well microtest plates. 30 μL of H_2_O_2_ solution was then added and NADPH oxidation into NAD^+^ was measured for 5 min by spectrophotometry at 340 nm.

Total SOD activity measurement was based on the higher affinity of SOD for superoxide anion (O_2_^•−^) than nitrobluetretrazolium (NTB), producing detectable blue formazan. 250 μL of a solution containing NTB, trizmahydrochloride, diethylenetriaminepentaacetic acid and hypoxanthine was added to 20 μL of plasma in 96-well microtest plates. 20 μL of xanthine oxidase (1.02U·mL^−1^) was then added and reacted with hypoxantine to produce O_2_^•−^. The appearance of blue formazan was measured by spectrophotometry at 560 nm for 5 min. SOD activity was computed by subtracting the rate of blue formazan appearance with deproteinized plasma (blank) to those with plasma sample.

#### Nitric oxide (NO) metabolites

Nitrite (NO_2_^−^) levels were detected by using 2,3-diaminonaphtalene (DAN) which fixes nitrite and emits at 450 nm after an excitation at 365 nm. 18 μL of DAN solution containing DAN and HCl was added to 10 μL of plasma diluted in 90 μL of H_2_O in 96-well microtest plates. After 10 min the reaction was inhibited with 18 μL oh NaOH solution. NO_2_- levels was measured by fluorometry (excitation at 365 nm and emission at 450 nm) and computed with NO_2_ standards.

To measure total NO_2_- and nitrate (NOx) levels, nitrate was reduced into NO_2_-, and NO_2_- was then measured as described above. 40 μL of nitrate reductase solution was added to 10 μL of plasma in 96 well microplates and 15 min later 50 μL of H_2_O was added. Then NO_2_- level is determined as described above.

### Statistical analyses

The required sample size of fifteen preterm and fifteen term born adults was established a priori (α = 0.05, β = 0.80) based on previous data from others^21^ and by our research group^25^ investigating microvascular reactivity and oxidative stress responses, respectively, in different populations.

All data are presented as mean ± SEM throughout the manuscript. A two-way (group × condition) repeated measures ANOVA was performed to compare changes in microvascular and oxidative stress responses during acute exposure to altitude between preterm and term born adults. Significant interaction effects were analyzed by Sidak correction. After having checked for normality using the Shapiro–Wilk normality test, pulmonary function, and percent changes from sea-level to high-altitude in preterm and term born individuals were compared by independent student’s t-test. Linear regression and correlation analyses were carried out by the least-squared residuals method. All *p*-values are two-tailed and statistical significance was defined a priori at *p* < 0.05. Data analyses were performed using the statistical software package Prism v.6.0 (GraphPad Software, San Diego, CA, USA).

## Results

Participants’ physical characteristics are reported in Table 1. Participants’ spirometry and lung diffusion capacity to carbon monoxide were presented in Manferdelli et al.^4^. No difference was observed between the two groups.

**Table 1:** Participants’ physical characteristics (Mean ± SEM).

	Term born	Preterm	*P*-value
Participants' characteristics			
Gestational age (weeks)	40 ± 0	29 ± 1	*P* < 0.001
Birth weight (g)	3621 ± 101	1132 ± 64	*P* < 0.001
Age (years)	21 ± 1	21 ± 1	*P* = 0.490
Height (cm)	182 ± 2	178 ± 2	*P* = 0.210
Body mass (kg)	75.6 ± 1.7	72.4 ± 3.5	*P* = 0.415
Body mass index (kg·m^−2^)	22.8 ± 0.4	22.5 ± 0.7	*P* = 0.713
Body surface area (m^2^)	1.95 ± 0.03	1.89 ± 0.06	*P* = 0.322
V̇O_2peak_ (mL·min^−1^·kg^−1^)	51.9 ± 1.9	48.5 ± 2.6	*P* = 0.290

Systolic blood pressure was similar between groups (preterm: 128 ± 2 mmHg; term born: 127 ± 2 mmHg, *p* = 0.633) and conditions (sea-level: 128 ± 2 mmHg; high-altitude: 127 ± 2 mmHg, *p* = 0.571), while a main effect of condition was found for diastolic blood pressure (sea-level: 75 ± 2 mmHg; high-altitude: 79 ± 1 mmHg, *p* < 0.001).

At sea-level, ATT at the site of the NIRS probe was similar between prematurely born (4.8 ± 0.6 mm) and term born (4.8 ± 0.4 mm, *p* = 0.983) adults and it was not affected by acute exposure to high-altitude in both preterm (4.8 ± 0.5, *p* = 0.797) and term born (4.5 ± 0.4, *p* = 0.122) participants.

### NIRS-VOT-derived parameters of muscle oxygenation and reactivity

**Figure 1:**
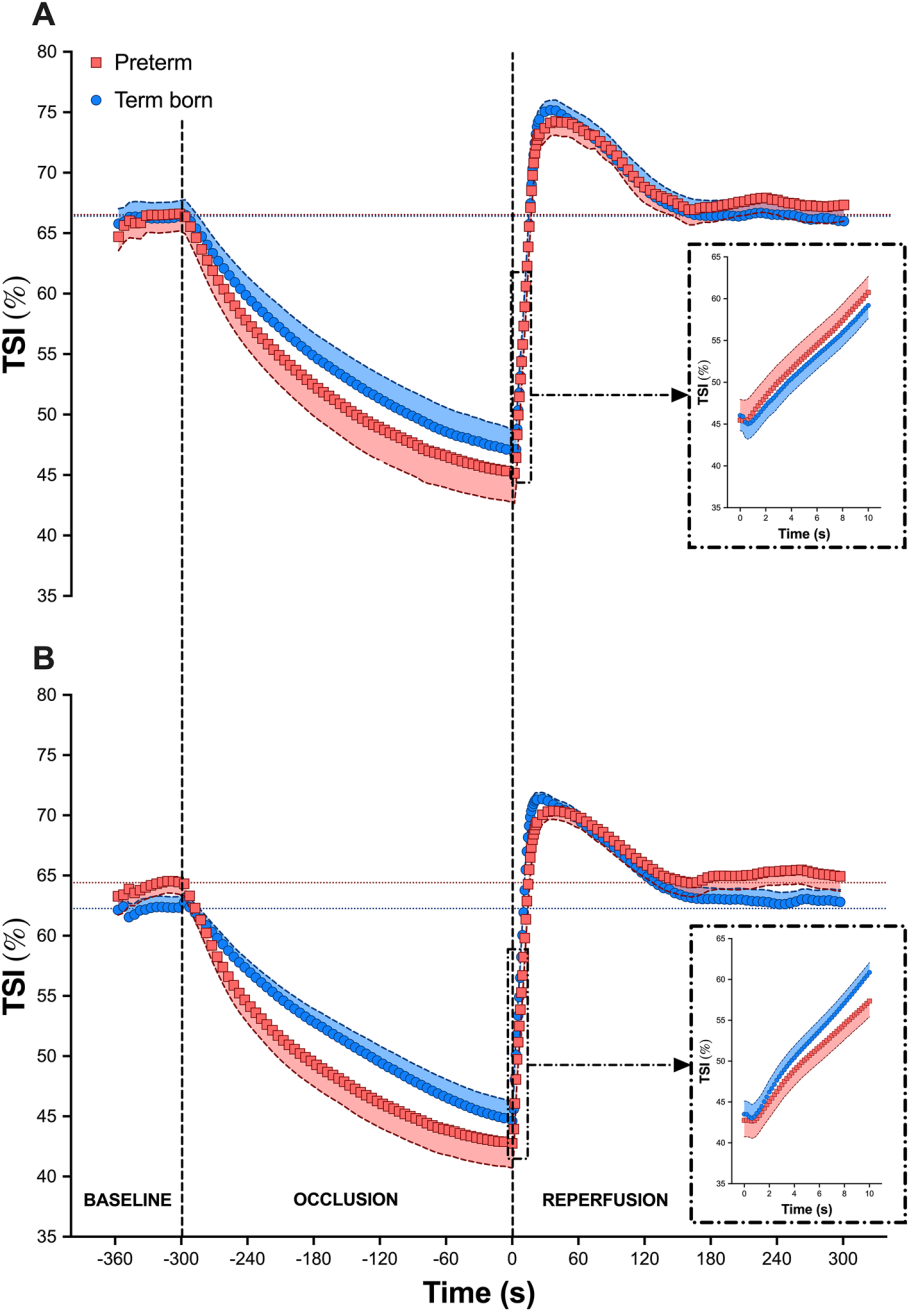
Groups mean (± SEM) of oxygen saturation signal (TSI) during the vascular occlusion test (VOT) performed at sea-level (panel **A**) and during acute exposure to high-altitude (3375 m; panel **B**) in prematurely born (red squares) and term born (blue circles) adults. Vertical dashed lines indicate instances of cuff inflation and deflation, while horizontal dashed lines indicate baseline values for each group. For each condition, the lower panel on the right side depicts the mean data during the first 10 s after cuff release (reperfusion rate).

**Figure 2:**
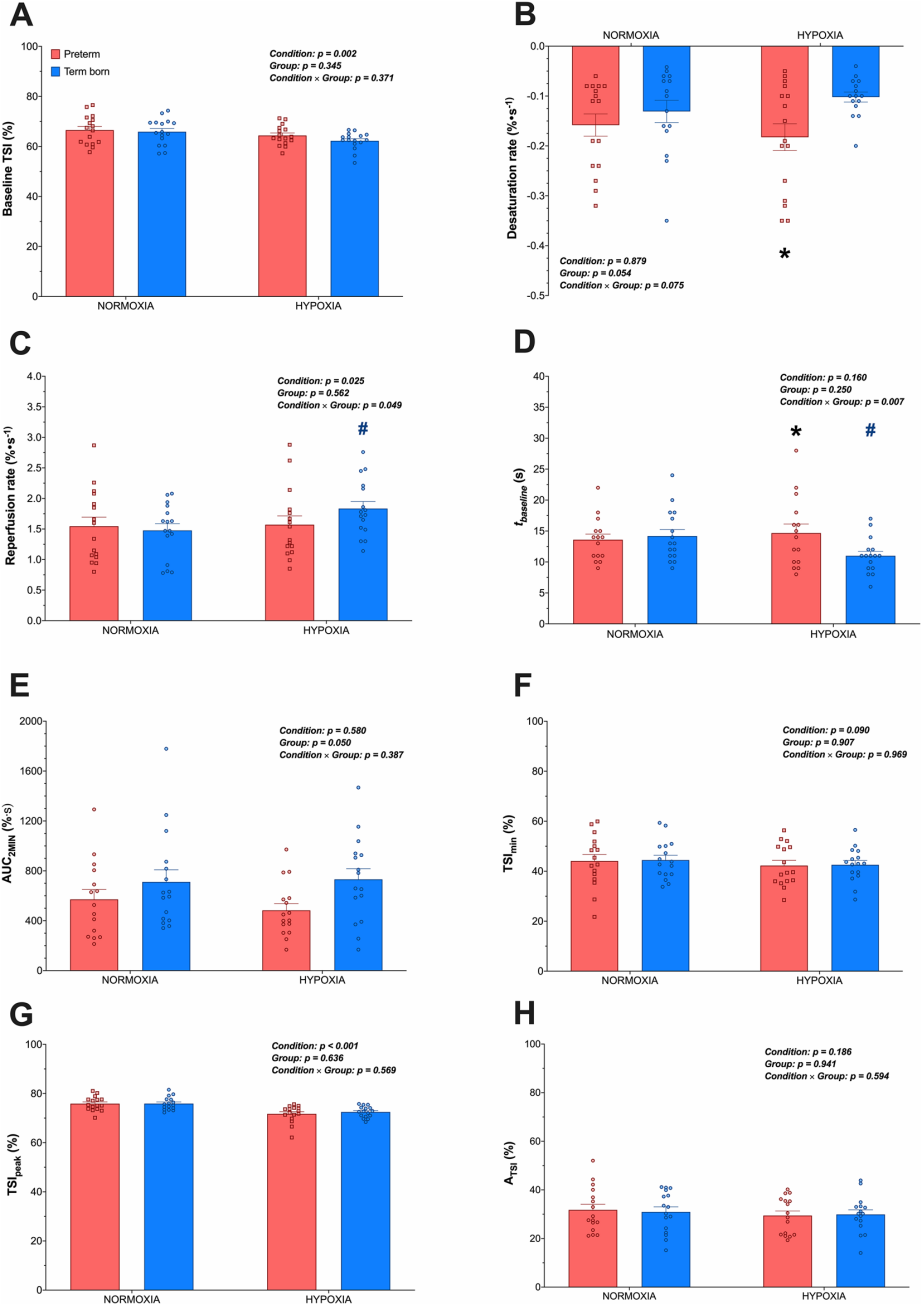
NIRS-VOT derived parameters (*panel ****A***, baseline TSI; *panel ****B***, desaturation rate; *panel ****C***, reperfusion rate; *panel ****D***, time to baseline (*t*_*baseline*_); *panel ****E***, area under the curve above baseline value during the first 2 min after cuff release (AUC_2MIN_); *panel ****F***, minimum TSI value reached during the occlusion phase (TSI_min_); *panel ****G***, peak TSI value reached during the occlusion phase (TSI_peak_); *panel ****H***, amplitude of TSI change during the reperfusion phase (A_TSI_) in prematurely born (red squares) and term born (blue circles) participants at sealevel and during acute exposure to altitude (3375 m). Data are expressed as Mean ± SEM. *Significantly different from term born; #Significantly different from normoxia.

Average TSI dynamics for both groups at sea-level and at high-altitude are shown in Fig. 1A, B, respectively. NIRS-VOT-derived parameters are reported in Fig. 2. Interestingly, the reperfusion rate did not change from sea-level to high-altitude in preterm (1.54 ± 0.15% s^−1^ vs. 1.57 ± 0.14% s^−1^, *p* = 0.969) participants while it increased in term born (1.48 ± 0.11% s^−1^ vs. 1.83 ± 0.12% s^−1^, *p* = 0.008) adults. Similarly, *t*_*baseline*_ was similar at high-altitude compared to sea-level in preterm (13.6 ± 0.9 s vs. 14.7 ± 1.5 s, *p* = 0.539) adults, while it was faster in term born (14.2 ± 1.0 s vs. 11.0 ± 0.7 s, *p* = 0.008) participants. In turn, *t*_*baseline*_* was* significantly slower in preterm compared to term born participants upon acute high-altitude exposure (*p* = 0.035).

### Skeletal muscle oxidative capacity

**Figure 3:**
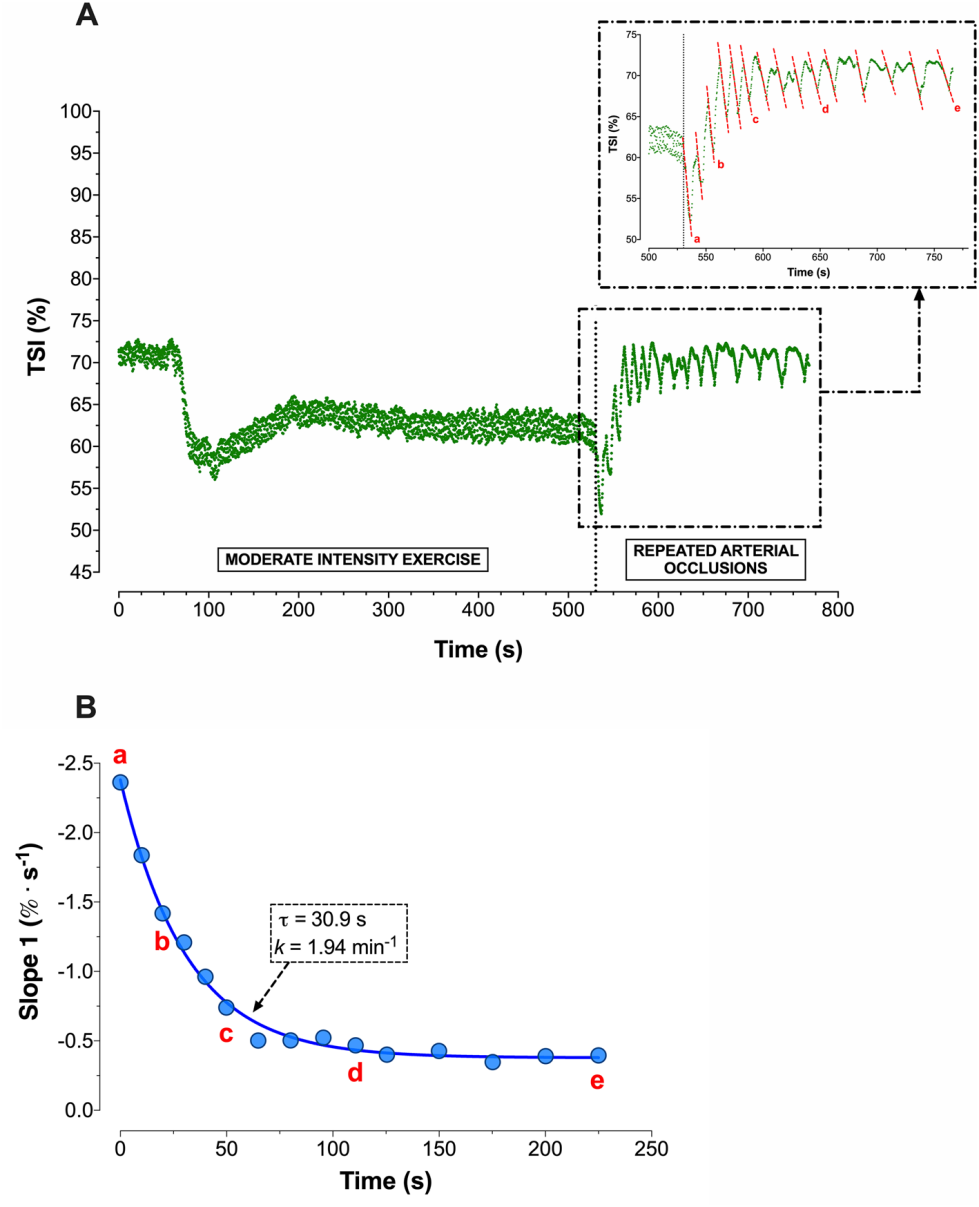
Representative participant (*term born, normoxia*) response during the muscle oxidative capacity assessment. Panel (**A**) illustrates the TSI dynamics during moderate intensity exercise followed by a series of intermittent arterial occlusions during recovery. The panel on the upper right corner shows the TSI changes during intermittent arterial occlusions and the red dotted lines represent the linear regression during each occlusion. Panel (**B**) depicts the slopes of each occlusion and the calculated muscle V̇O_2_ (*m*V̇O_2_) recovery profiles (dashed line). *k* represents the rate constant, which is linearly related to muscle oxidative capacity (k = (1/τ) 60, min^−1^. The letters (a–e) illustrate how the corresponding *m*V̇O_2_ value is derived from respective TSI negative slopes during intermittent occlusions.

**Figure 4:**
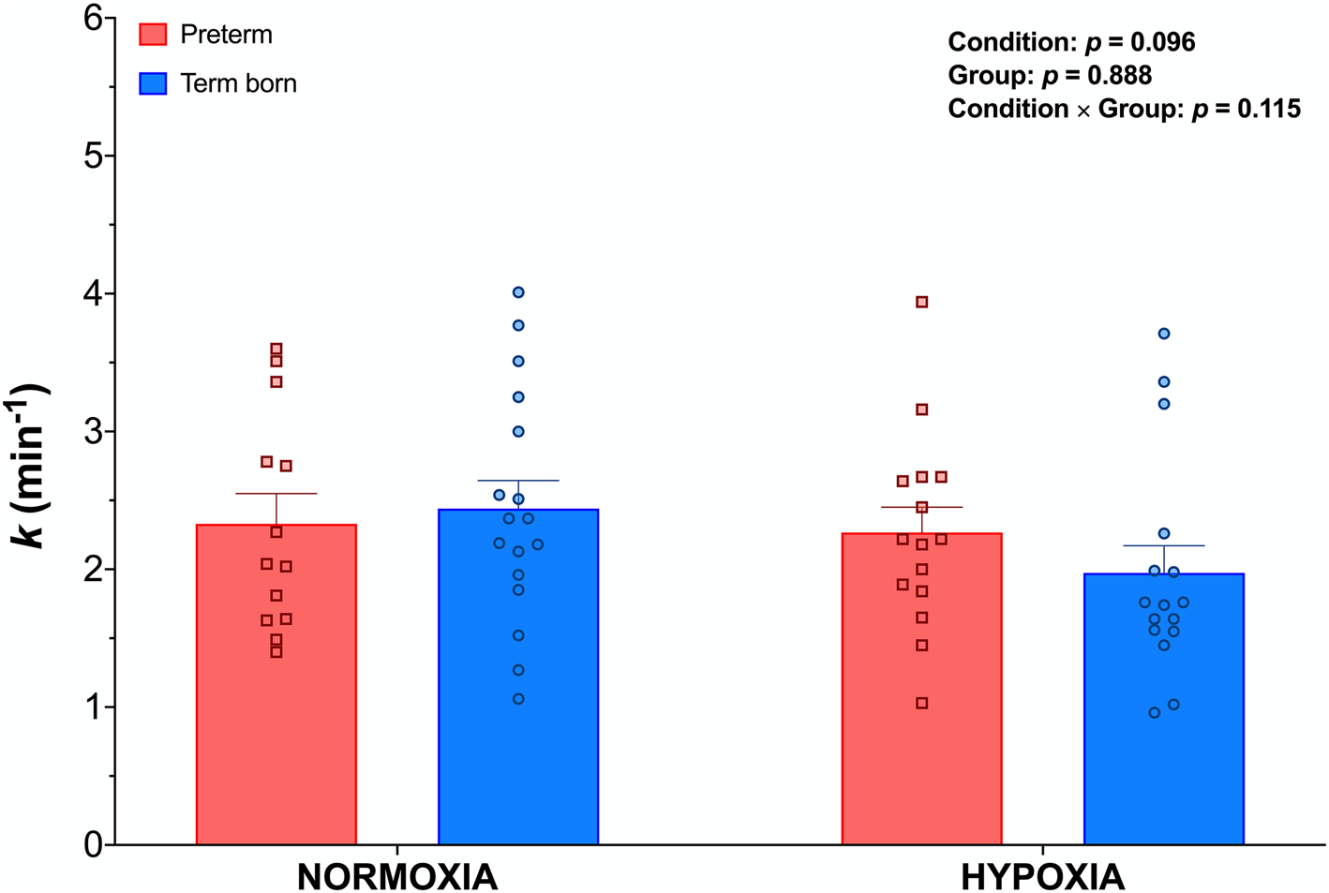
Groups mean (± SEM) of skeletal muscle V̇O_2_ recovery rate constant (*k*) at sea-level and during acute exposure to high-altitude (3375 m) in prematurely born (red squares) adults and their term born (blue circles) peers. *Significantly different from term born.

A graphical representation of TSI changes during the repeated arterial occlusion protocol in a typical participant (*term born, normoxia*) is shown in Fig. 3. A similar *k* was found between groups both at sea-level (*p* = 0.795) and during acute exposure to high-altitude (*p* = 0.625; Fig. 4). DmO_2_ was significantly lower in preterm compared to term born participants (−0.3 ± 0.2 min^−1^ vs. 0.4 ± 0.1 min^−1^, *p* = 0.029).

### Oxidative stress

**Figure 5:**
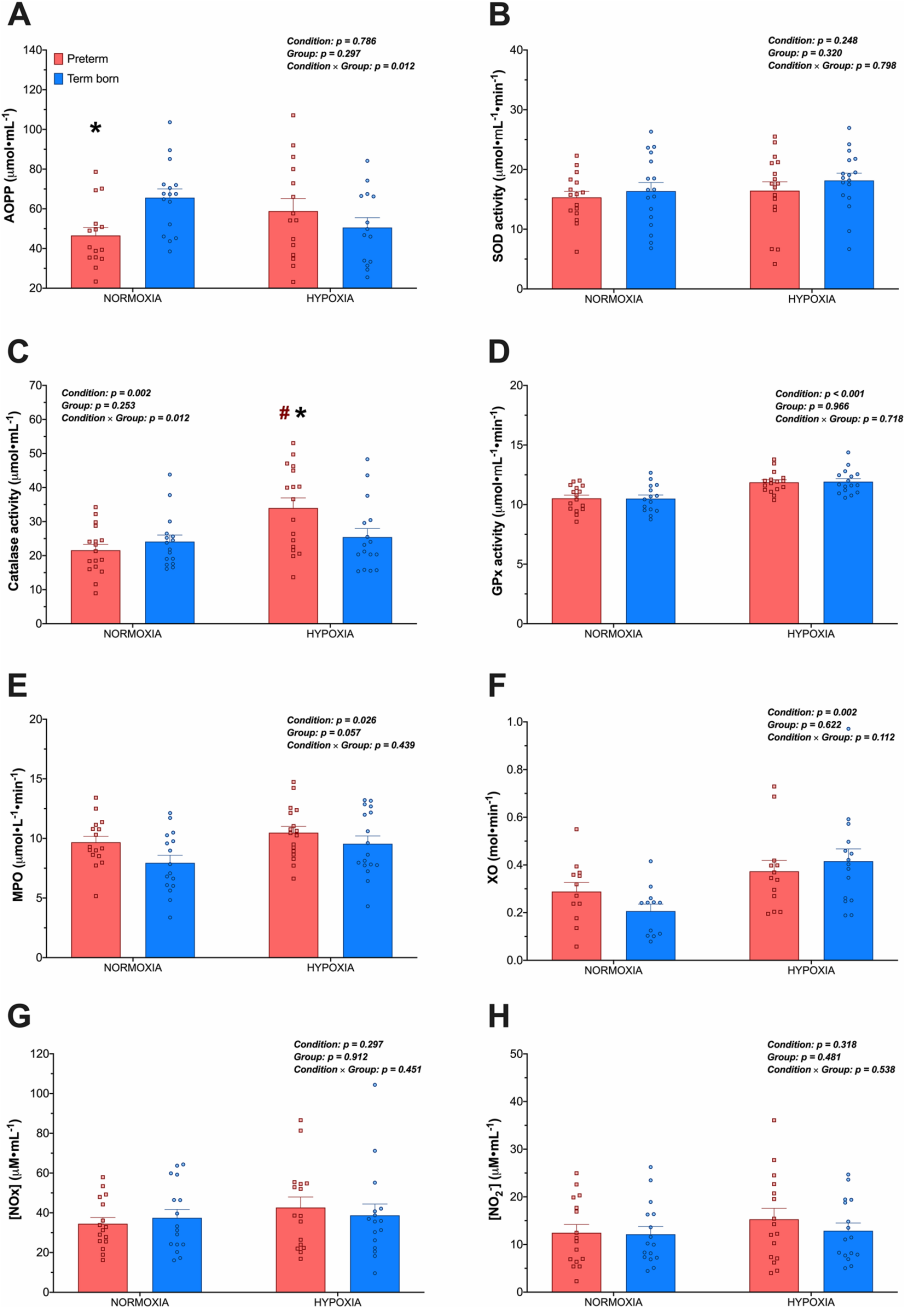
Groups mean (± SEM) of oxidative stress markers, antioxidant enzymes, and nitric oxide metabolites in plasma at sea-level and upon arrival at high-altitude (3375 m) in preterm (red squares) and term born (blue circles) adults. AOPP, advanced protein oxidation products (panel **A**); SOD, superoxide dismutase (panel **B**); catalase activity (panel **C**); GPx, glutathione peroxidase (panel **D**); MPO, Myeloperoxidase (panel **E**); XO, xanthine oxidase (panel **F**); NOx, total nitrite and nitrate (panel **G**); NO_2_^−^, nitrite (panel **H**). *Significantly different from term born; #Significantly different from normoxia.

Oxidative stress markers, antioxidant enzyme and NO metabolites at sea-level and at high-altitude in both groups are reported in Fig. 5. The altitude-induced increase in plasma XO was significantly lower in preterm compared to term born participants (28.7 ± 26.0% vs. 159.3 ± 48.7%, *p* = 0.030). In contrast, preterm adults showed a more pronounced altitude-induced increase in plasma AOPP and catalase (35.3 ± 61.0% and 66.9 ± 63.9%, respectively) compared to term born adults (-13.2 ± 13.2% and 15.2 ± 16.9%, *p* = 0.034 and *p* = 0.010, respectively).

### Correlations

At sea-level, the reperfusion rate was significantly correlated with *t*_*baseline*_ in both preterm (*r* = −0.591, *p* = 0.020) and term born (*r* = −0.567, *p* = 0.022) adults. Likewise, *t*_*baseline*_ was significantly correlated with NO_2_^−^ levels in preterm (*r* = −0.559, *p* = 0.038) but not term born participants (*p* = 0.135). Moreover, diastolic blood pressure was correlated with the reperfusion rate in preterm (*r* = 0.611, *p* = 0.012), but not in term born participants (*p* = 0.588).

## Discussion

The aim of this study was to investigate the peripheral (microvascular reactivity and skeletal muscle oxidative capacity) and systemic oxidative stress responses to acute high-altitude exposure in prematurely born but otherwise healthy adults and in their term born peers. To the best of our knowledge, this is the first study to report a blunted post-occlusive reactivity at the microvascular level and higher skeletal muscle oxidative capacity during acute exposure to high-altitude in adults born preterm compared to term born participants. Also, prematurity appeared to confer specific altitude-induced changes in some pro-oxidant/antioxidant markers.

The microvascular responsiveness was assessed by monitoring NIRS-derived TSI changes in the *vastus lateralis* muscle during VOT^14,15^. Under normoxic conditions, a blunted microvascular reactivity to a 5-min arterial occlusion was observed in patients with cardiovascular diseases^19^ and sedentary individuals^20,21^ compared to healthy and trained subjects, respectively. In addition, microvascular reactivity assessed by NIRS in the thenar muscles at different altitudes was reduced following a short period of ischemia (3 min) in resting skeletal muscle of acclimatizing healthy adults compared to sea-level^26^. In the present study, partly supporting our second hypothesis, acute high-altitude exposure did not induce any change in reperfusion rate or muscle oxygenation *t*_*baseline*_ in preterm adults, while these responses were altered in term born individuals. Accumulating evidence suggests that acute exposure to hypoxia induces release of pro-angiogenic factors and local vasodilators (i.e., NO, adenosine, acidosis), leading to capillary recruitment and increased microcirculatory oxygen extraction capacity^27^. It is therefore reasonable to speculate that skeletal muscle microvascular responsiveness was improved in term born individuals, likely due to an increased number of perfused capillaries during acute exposure to altitude. In contrast, the microvascular response was unchanged in preterm individuals, suggesting a blunted microcirculation response to acute exposure to high-altitude in this group. Our findings may therefore support previous results on lower microvascular function and density in healthy adults born prematurely^3^. Ultimately, this impaired physiological response may mechanistically underpin the potentially increased susceptibility of these individuals to high-altitude illnesses, in particular to high-altitude pulmonary and cerebral edema^28^. An important methodological point when assessing vascular responsiveness to VOT is the potential influence of the ischemic stimulus on the magnitude of the response (reperfusion)^14^. Given that high-altitude exposure decreased baseline TSI similarly in both groups (~ 4%), and that both term born and preterm participants reached similar TSI values at the end of the occlusion period, the differences observed in post-occlusive reperfusion in this study are unlikely to be due to a different ischemic stimulus.

The present study also investigated skeletal muscle oxidative capacity and DmO_2_ using NIRS-derived TSI during the repeated occlusions method^16^. Even though acute hypoxia is known to impair mitochondrial function^10^, the altitude-induced decrease in oxidative capacity was significantly lower in our preterm cohort compared to their term born peers. Increased mitochondrial oxygen consumption was observed in peripheral blood mononuclear cells of prematurely born adults^13^. In contrast, postnatal hyperoxic exposure in male rats was shown to reduce mitochondrial function relative to a control group provided with no treatment^12^. Despite these recent findings, oxidative function at the skeletal muscle level remains significantly under-investigated in this population. In fact, several comorbidities typically described in adult survivors of premature birth are characterized by increased mitochondrial dysfunction and chronic oxidative stress in non-preterm populations. To the best of our knowledge, we are the first to report a lower susceptibility to altitude-induced decreases in skeletal muscle oxidative capacity in preterm adults. However, further research is warranted to elucidate the underlying mechanisms of this specific response to acute high-altitude/hypoxia in this cohort.

Furthermore, DmO_2_ assessed as recently proposed^18,24^ was lower in preterm adults compared to their born term peers. Although influenced by many other factors, DmO_2_ is proportional to capillary density^29^, and represents one of the key determinants of exercise capacity in humans^30^. However, a recent study published by our group demonstrated that exercise capacity in preterm adults—with similar relative V̇O_2peak_ but lower peak power output compared to term born peers—is primarily impaired by convective rather than diffusive O_2_ transport mechanisms^4^. Despite this, peripheral skeletal muscle limitations have recently been suggested to be an important underlying mechanism limiting exercise capacity in adults born preterm^31^. The present findings of reduced DmO_2_ are in line with previous results from animal models^12^ and suggest skeletal muscle specific long-term prematurity sequelae.

Finally, exposure to hypoxia increases oxidative stress in the general term born population^25^. The present study also investigated NO metabolites and oxidative stress markers in response to acute exposure to high-altitude. Increased oxidative stress in normoxic conditions was reported in preterm infants due to their immature defense systems against reactive oxygen species (ROS), immature organs and the need for medical treatments to increase ROS production^10^. In this population, higher oxidative stress levels persist through young adulthood and the subsequent redox imbalance might result in a ‘preconditioning’ state, or it may be involved in the pathogenesis of several non-communicable chronic diseases^10^. However, the present study did not find an increased oxidative stress in normoxia in prematurely born adults. Our contrasting results might be explained by the relatively high V̇O_2peak_ of our preterm cohort compared to the values typically reported in the literature for this population^32^. However, a lower altitude-induced increase in XO and higher levels of both AOPP and catalase in response to acute high-altitude exposure were observed in preterm compared to term born participants, suggesting an increased systemic H_2_O_2_ production upon arrival to high-altitude in preterm compared to term born adults. Likewise, the lower hypoxia-induced increase in XO during acute high-altitude exposure in prematurely born adults may be an adaptive mechanism in addition to the increased catalase activity. A recent comprehensive review on the adaptive mechanisms to hypoxic exposure highlighted mitochondria and activation of nicotinamide adenine dinucleotide phosphate oxidase as main sources of ROS formation in humans^33^. Although systemic oxidative stress may not be reflective of solely muscle oxidative stress only, skeletal muscles represent the main source of oxidative stress, particularly in under combined hypoxia and exercise^34,35^. Therefore, it could be hypothesized that a greater skeletal muscle oxidative capacity should result from an upregulated mitochondrial function. In turn, the exaggerated mitochondrial function in preterm recently reported in this population^13^, could lead to an even greater release of reactive oxygen species from the muscle and explain their increased systemic oxidative stress. Therefore, the increased ROS formation in this study, in conjunction with higher skeletal muscle oxidative capacity during acute high-altitude exposure, suggest an upregulated mitochondrial function in preterm individuals compared to their term born peers under conditions of reduced oxygen availability. In contrast, NOx and NO_2_^−^ were not affected by exposure to high-altitude in both groups. While NO is undoubtedly involved in the pulmonary, cardiovascular, and muscular responses to high-altitude exposure^36^, a recent well-conducted study demonstrated that NO metabolites peak after 3 days at an altitude similar to that used in the present study. Furthermore, the present study did not observe a direct correlation between plasma NO_2_^−^ and NOx concentrations and microvascular responsiveness at sea-level in term born adults. Despite the central role of NO on both macro and microvascular function^37^, it seems that other active signaling molecules and/or patterns (e.g., PGI_2_, Adenosine, K_ATP_ channels) regulated by tissue hypoxia concomitantly regulate vasodilation processes and muscular perfusion^14^. On the contrary, *t*_*baseline*_ was inversely correlated with NO_2_^−^ levels in prematurely born participants, suggesting that microvascular function in this population is primarily driven by NO metabolites at sea-level.

### Methodological considerations

Even though the present study was the first to assess microvascular responsiveness and in vivo skeletal muscle oxidative capacity in prematurely born adults at both sea-level and acute high-altitude exposure, we would like to acknowledge a few important limitations. First, our prematurely born cohort had a relatively high V̇O_2peak_ and normal pulmonary function compared with those generally reported by others^32^. Therefore, the relatively active preterm adults investigated in this study may not ideally represent typical preterm born adults. However, according to recent findings^38^, this might be a consequence of them being born at the turn of the millennium with better neonatal management and consequently improved long-term pulmonary outcomes. Given the continued advancements in neonatal medicine, studies of preterm birth survivors receiving more contemporary treatments are (and will continue to be) of increasing importance. In addition, the present study investigated only male survivors of preterm birth, despite existing evidence in rodent studies that sex differences likely exist in the association between preterm birth and skeletal muscle physiology^12^. We acknowledge the need for further research on the long-term sequelae of premature birth in female participants. Finally, one may question whether the compensatory mechanisms observed in this study were maximised in the preterm born group. Unfortunately, the present study was unable to quantify this response in relation to the individual capacity to adapt to high altitude. This could however be a particularly interesting avenue for future research.

### What is the clinical relevance of the present findings?

A growing body of literature suggests a specific phenotypical cardiovascular response to hypoxia in this cohort^4,32,39^. While some authors hypothesized an increased risk of right ventricular failure due to an exaggerated cardiac contractile response to hypoxia, our findings seem to suggest an unresponsiveness of the microcirculation to acute exposure to high-altitude in preterm adults. Intriguingly, a blunted microvascular responsiveness was suggested to represent an early sign for later macrovascular dysfunction^40^. It seems therefore that hypoxia may represents a risk factor for the cardiovascular system in this population. However, we recommend further studies before drawing conclusions of clinical relevance.

## Conclusion

Overall, this study provides novel insights into the peripheral (microvascular responsiveness and skeletal muscle oxidative capacity) and oxidative stress responses to acute high-altitude exposure in adults born prematurely. Preterm adults showed higher oxidative stress and blunted peripheral responses to acute exposure to high-altitude compared to term born participants, as demonstrated by unchanged microvascular reactivity and skeletal muscle oxidative capacity. This observed blunted response to acute high-altitude exposure observed in prematurely born (but otherwise healthy) adults may compromise altitude acclimatization and ultimately lead to an increased risk of high-altitude illnesses. Future research is necessary to substantiate our findings and should focus on measuring skeletal muscle diffusion and oxidative capacity using direct methods both at rest and during exercise, as well as under different environmental conditions such as hypoxia or hypobaria.

## Data Availability

The data that support the findings of this study are available from the corresponding author upon reasonable request.
